# Generation of a Prophage-Free Variant of the Fast-Growing Bacterium Vibrio natriegens

**DOI:** 10.1128/AEM.00853-19

**Published:** 2019-08-14

**Authors:** Eugen Pfeifer, Slawomir Michniewski, Cornelia Gätgens, Eugenia Münch, Felix Müller, Tino Polen, Andrew Millard, Bastian Blombach, Julia Frunzke

**Affiliations:** a Forschungszentrum Jülich GmbH, Institute for Bio- and Geosciences 1, IBG1, Jülich, Germany; b Warwick Medical School, University of Warwick, Coventry, United Kingdom; c Department Genetics and Genome Biology, University of Leicester, Leicester, United Kingdom; d Institute of Biochemical Engineering, University of Stuttgart, Stuttgart, Germany; e Microbial Biotechnology, Campus Straubing for Biotechnology and Sustainability, Technical University of Munich, Straubing, Germany; University of Georgia

**Keywords:** spontaneous prophage induction, *Vibrio*, bacteriophages, genome reduction, prophage, prophage-free, stress response

## Abstract

The fast-growing marine bacterium Vibrio natriegens represents an emerging strain for molecular biology and biotechnology. Genome sequencing and quantitative PCR analysis revealed that the first chromosome of *V. natriegens* ATCC 14048 contains two prophage regions (VNP1 and VNP2) that are both inducible by the DNA-damaging agent mitomycin C and exhibit spontaneous activation under standard cultivation conditions. Their activation was also confirmed by live cell imaging of an mCherry fusion to the major capsid proteins of VNP1 and VNP2. Transmission electron microscopy visualized the release of phage particles belonging to the *Siphoviridae* family into the culture supernatant. Freeing *V. natriegens* from its proviral load, followed by phenotypic characterization, revealed an improved robustness of the prophage-free variant toward DNA-damaging conditions, reduced cell lysis under hypo-osmotic conditions, and an increased pyruvate production compared to wild-type levels. Remarkably, the prophage-free strain outcompeted the wild type in a competitive growth experiment, emphasizing that this strain is a promising platform for future metabolic engineering approaches.

**IMPORTANCE** The fast-growing marine bacterium Vibrio natriegens represents an emerging model host for molecular biology and biotechnology, featuring a reported doubling time of less than 10 minutes. In many bacterial species, viral DNA (prophage elements) may constitute a considerable fraction of the whole genome and may have detrimental effects on the growth and fitness of industrial strains. Genome analysis revealed the presence of two prophage regions in the *V. natriegens* genome that were shown to undergo spontaneous induction under standard cultivation conditions. In this study, we generated a prophage-free variant of *V. natriegens*. Remarkably, the prophage-free strain exhibited a higher tolerance toward DNA damage and hypo-osmotic stress. Moreover, it was shown to outcompete the wild-type strain in a competitive growth experiment. In conclusion, our study presents the prophage-free variant of *V. natriegens* as a promising platform strain for future biotechnological applications.

## Abstract (precis)

The fast-growing marine bacterium Vibrio natriegens represents an emerging model host for molecular biology and biotechnology, featuring a reported doubling time of less than 10 minutes. In many bacterial species, viral DNA (prophage elements) may constitute a considerable fraction of the whole genome and may have detrimental effects on the growth and fitness of industrial strains. Genome analysis revealed the presence of two prophage regions in the *V. natriegens* genome that were shown to undergo spontaneous induction under standard cultivation conditions. In this study, we generated a prophage-free variant of *V. natriegens*. Remarkably, the prophage-free strain exhibited a higher tolerance toward DNA damage and hypo-osmotic stress. Moreover, it was shown to outcompete the wild-type strain in a competitive growth experiment. In conclusion, our study presents the prophage-free variant of *V. natriegens* as a promising platform strain for future biotechnological applications.

## INTRODUCTION

Bacteriophages, or phages, represent the most abundant biological entity on Earth (1) and feature highly diverse lifestyles. Temperate phages are able to integrate into the bacterial genome, where they maintain, as so-called prophages, a long-term association with their host. This status fosters mutual adaptation between the host and the viral genome, and bioinformatic studies revealed that prophages and phage remnants can make up 20% of an entire bacterial genome (2, 3). Prophages are found in nearly all bacterial genomes and are the cause for the majority of strain-specific differences within a bacterial species (4–7). In recent years, more and more studies focused on resolving the impact of the prophages on host physiology (8–11). However, stressful conditions causing, e.g., DNA damage can reactivate the prophage, which usually leads to a switch to the destructive program of the phage (i.e., lytic cycle) and ultimately to cell lysis and the release of phage particles. Stochastic effects such as fluctuations of repressor molecules or spontaneously occurring single-stranded DNA (e.g., triggered by stalled replication forks) or strand breaks may provoke a phenomenon termed spontaneous prophage induction (SPI) (12). Although SPI is the cause of a constant loss of individuals in a bacterial population, several studies emphasized its positive impact on the fitness of host bacteria (13, 14), for example, by increasing biofilm formation (15) or virulence by lysis-dependent toxin release (16).

In industrial fermentation processes, the induction of prophages represents a consequent loss of producer cells and may cause severe economic losses. Thus, many strain optimization efforts as well as genome reduction projects have focused on the removal of prophages and prophage-like regions from the genome (17–22). In the majority of the studies it was shown that under laboratory conditions the prophage-free variants are more robust and better able to be harnessed as microbial cell factories (17, 19, 21, 23). In general, microbial production processes are optimized to achieve high space-time yields and, thus, the growth rate of a microorganism often represents a key limiting factor. This is simply due to the fact that fast-growing producers will consume more substrate (i.e., possess a high-biomass-specific substrate consumption rate) and, therefore, have the potential to achieve higher productivity than slow-growing producers. Consequently, there is a strong interest in fast-growing, nonpathogenic, and robust microorganisms featuring high substrate uptake rates to improve biotechnological processes (24).

The fast-growing, Gram-negative bacterium Vibrio natriegens represents a promising candidate with a high potential to speed up biotechnological processes. Isolated in 1958 from a salt marsh mud region, the first studies reported on doubling times of less than 10 min (25, 26). Intrigued by the fast growth and high substrate uptake rate, several recent studies focused on the potential of *V. natriegens* as a host for molecular biology, including cloning approaches (27, 28), protein synthesis (28–31), and small-molecule production (24, 32).

In this work, we focused on the construction and characterization of a prophage-free variant of *V. natriegens* ATCC 14048. Two potential intact prophage regions were first predicted *in silico* and ultimately confirmed by mitomycin C induction experiments. In further experiments, the two prophages (termed VNP1 and VNP2) were removed from the bacterial genome. Comparative analysis revealed that cells lacking the two prophages were more robust under hypo-osmotic stress as well as conditions causing DNA damage. Moreover, in a competitive growth experiment, the prophage-free variants outcompeted the wild type (WT), especially under hypo-osmotic conditions. This could be attributed to a continuous loss of a small fraction of the wild-type population due to SPI. Overall, our data emphasized that the prophage-free *V. natriegens* strain is a promising platform for future metabolic engineering and biotechnological applications.

## RESULTS

### Prophages in Vibrio natriegens genomes.

**FIG 1:**
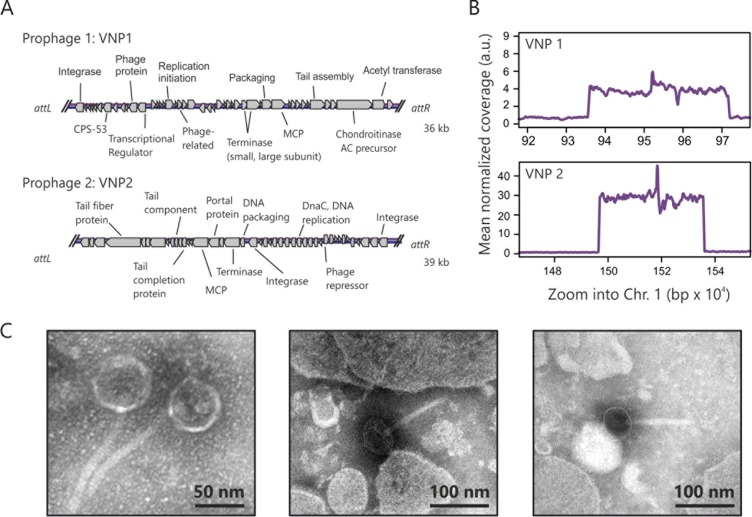
Inducible prophages in the genome of *V. natriegens* ATCC 14048. (A) In the genome of ATCC 14048 (Assembly no. GCA_001456255.1), two prophage regions were predicted by PHASTER (34) and PihSpy (33); both are located on chromosome 1 (Chr. 1). The annotation is based on the latest RefSeq sequence and RAST (35). All coding sequences that do not encode hypothetical proteins are highlighted and indicated by lines. (B) Addition of 1 μM MMC to a *V. natriegens* culture (after at least 4 h of preincubation) resulted in a significant enrichment of prophage regions, as verified by sequencing of DNA in the culture supernatant. a.u., arbitrary units. (C) Moreover, phage particles were also confirmed by transmission electron microscopy showing the presence of phages from the *Siphoviridae* family.

Currently, in the NCBI RefSeq database complete genomes sequences of five different *V. natriegens* strains are available (8 April 2019). Each genome consists of two bacterial chromosomes, which is a typical feature of *Vibrio* species. The ATCC 14048 isolate was previously described as one of the fastest-growing strains among the currently known *V. natriegens* strains (28). It is noteworthy that different isolates of ATCC 14048 are available. In this work, we focused on the strain with BioSample accession number SAMN03178087. Using PhiSpy (33) and PHASTER (34), two prophage regions were predicted in the first chromosome (RefSeq accession number NZ_CP009977) (see Fig. S1 in the supplemental material). According to the PHASTER criteria, one of the prophages (from PN96_RS04340 to PN96_RS04580) is categorized as an incomplete phage and the other one (PN96_RS06975 to PN96_RS07040) as an intact phage. In addition to the latest RefSeq annotations, we used RAST (35) to extend the annotation of the potential prophages and found the presence of typical phage genes (e.g., integrases, tail, and major capsid proteins [MCP]) (Fig. 1A and Table S1). Moreover, PhiSpy predicted a third prophage region (PN96_RS22545 to PN96_RS22650) in the second chromosome (Fig. S1). However, in this particular region, no typical phage genes were predicted by either RAST or RefSeq, and several genes are annotated to encode proteins with typical bacterial functions, such as F_o_F_1_ ATP synthase subunits, ATP synthases, and an aldo/ketoreductase (Table S1). To test whether the prophages are still inducible, we added mitomycin C (MMC), a DNA cross-linking and damaging agent, to an exponentially growing wild-type culture. After 3 to 4 h the liquid culture turned cloudy and mucous, indicating cell lysis. DNA isolated from the supernatant was sequenced and revealed a specific enrichment of the two prophage regions of the first chromosome (Fig. 1B). No enriched DNA was found for the PhiSpy predicted prophage region of the second chromosome (data not shown). We named the two inducible prophages VNP1 and VNP2 (for Vibrio natriegens prophages 1 and 2). The enriched DNA amount of VNP2 (predicted as intact prophage according to PHASTER) was about seven times higher than that of VNP1, suggesting that VNP2 features a higher burst size. Derived from the sequencing data, we determined the exact length of VNP1 (36,053 bp), VNP2 (39,183 bp), and their attachment sites (*att*) [VNP1, 14 bp, TAGATTTGTGTGGT; VNP2, 26 bp, CAGCC(G/C)AC(A/T)TT(C/T)TTCTTCTTTGA(C/T)TA]. In the case of VNP2, we identified an impaired 26-bp-long *att* site. The sequencing revealed that the *attP* site, or rather, the sequence after induction (CAGCCGACATTCTTCTTCTTTGATTA), is slightly different from the *attL* (CAGCCCACTTTTTTCTTCTTTGATTA) and *attR* (CAGCCGACATTCTTCTTCTTTGACTA) sites before induction (differences in *att* sites are underlined). This is likely the result of the recombination event during excision of the phage genome from the bacterial chromosome.

Using transmission electron microscopy (TEM), we analyzed the supernatant of an MMC-induced wild-type sample and confirmed the presence of phages belonging to the *Siphoviridae* family. The particles consist of a head with an approximate diameter of 50 to 60 nm and a flexible, noncontractile tail length of 100 to 110 nm (Fig. 1C). Moreover, the assignment of the two phages to *Siphoviridae* was also predicted by Virfam (36), and no genes coding for sheath-forming proteins, which are typical for *Myoviridae*, were annotated in the prophage regions (Table S1).

### Distribution of VNP1- and VNP2-like elements in *Vibrio* species.

Using the nucleotide sequences of VNP1 and VNP2, a search for similar prophages in a *Vibrio* prophage database with more than 10,000 potential prophages was performed. The search resulted in 13 hits for VNP1-like and 77 hits for VNP2-like prophages according to a Mash similarity of at least 70%. Based on these genome similarities, hierarchical clustering was performed (Fig. S2 and S3). Three prophages grouped distinctly with VNP1 and, interestingly, one of these phages shows 100% identity, whereas the other two have an average nucleotide identity of ∼99%. Further detailed genome comparison revealed that the identities of the latter two mentioned phages are based on an ∼24-kb region covering ∼70% of the VNP1 region. Strikingly, an ~8-kb section of the remaining region in VNP1 shows an identity of 84% with a region within the VNP2 prophage, suggesting that the two prophages are capable of recombination. The prophage from Vibrio alginolyticus ZJ-T (SAMN05271497_p1) did not cluster with VNP1, but the Mash distance suggested some similarity between the prophage sequences, supported by an average nucleotide identity (ANI) of 91%. However, the comparison of the genome organizations revealed a lack of synteny and substantially different genome sizes, suggesting this prophage is a different phage species. ANI analysis of the remaining prophages showed a similarity of less than 85% that represents, according to current standards (9), a different phage species than VNP1 (Table S2).

Four phages clustered closely with VNP2. Three of these prophages were 100% identical to VNP2 and are predicted to be on the chromosomes that also contained the above-mentioned close derivatives of VNP1. The fourth prophage (SAMN02472057_p1) was assembled from several contigs and had an ANI of 96%, suggesting it is the same phage species as VNP2. Further genomic comparison identified a core set of 18 genes common to all five phages and a similar genome structure, but with the prophage from the CCUG 16373 isolate (SAMN05302750_p2) being ∼4 kb smaller (Table S2). The remaining prophages that were detected had an ANI of <90% with VNP2 and therefore represent different species. Strikingly, a few prophages that show a high ANI with VNP2 also share high Mash similarities with VNP1 (and vice versa) (Fig. S2 and S3 and Table S2). In conclusion, the comparative genomics analysis of VNP1 and VNP2 revealed that the two phages and their close relatives are specific to *V. natriegens* isolates but also shows that there is a greater distribution of the distantly related VNP2-like phages among *Vibrio* species.

### Curing *V. natriegens* from prophages.

**FIG 2:**
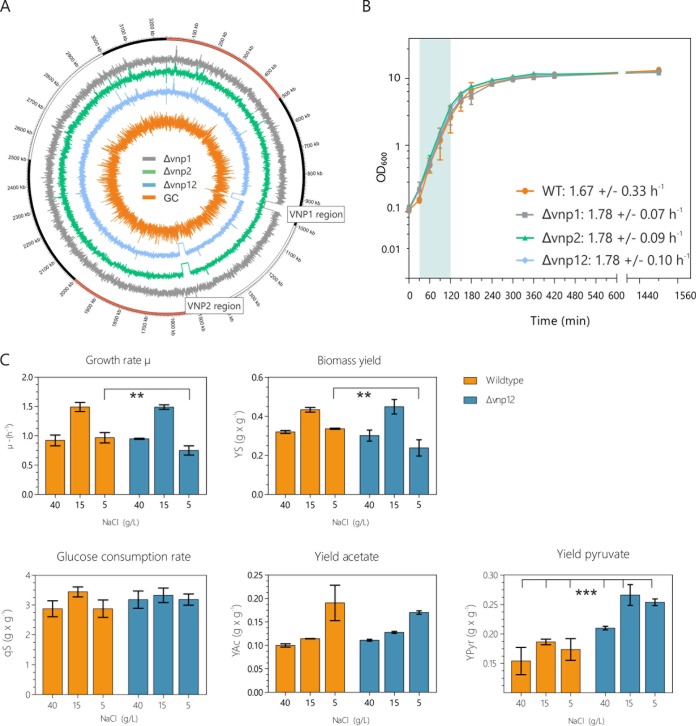
Impact of prophage deletion on growth and organic acid production. (A) Circular plot of the first chromosome from the prophage-free variants. The respective coverage tracks of the genome sequences of *Δvnp1*, Δ*vnp2*, and Δ*vnp12* strains were extracted and plotted clockwise using Circos (65). Curves pointing to the center of the plot indicate a drop in coverage, whereas an increase is indicated by curves pointing outwards. The orange line represents the GC content of the first chromosome of the *V. natriegens* strain ATCC 14048 (sliding window; step size, 150; window size, 300). Regions of VNP1 and VNP2 are highlighted. (B) Growth assay of the WT and the phage-free variants was performed in shaking flasks in BHIN at 30°C (*n* = 3 biological replicates; standard deviations are indicated by error bars). Indicated growth rates were calculated for the exponential growth phase, which is marked in the green area (30 to 150 min). (C) The specific glucose consumption rate (*q_S_*), the pyruvate, acetate, and biomass yield, and growth rate were determined for the wild-type (orange) and the phage-cured strain (blue). Cells were cultivated in the minimal VN media with 1% glucose and different sodium chloride concentrations (0.5%, 1.5%, and 4% [wt/vol], respectively). Values represent averages from three biological replicates; standard deviations are indicated by error bars. Significance was calculated using two-way analysis of variance comparing the means under the respective conditions and strains.

To remove the viral load from the genome of *V. natriegens*, we first deleted the VNP1 region by two-step homologous recombination and *sacB*-based counterselection. Although this method worked fine for VNP1, the second prophage could not be deleted. Hence, we used a different screening approach by integrating the *ccdB* toxin under the control of the P*_BAD_* promoter into the prophage region. The presence of the toxin led to the isolation of cells that have spontaneously lost the VNP2 region, as confirmed by PCR (data not shown) and genome sequencing (Fig. 2A). We left the *attB* sites in the *Vibrio* genome to enable later phage infection, transduction, and phage profiling experiments. Overall, the deletions resulted in a 2.3% reduction of the first chromosome. Moreover, genome sequencing revealed 16 single-nucleotide polymorphisms (SNPs), which were already present in the parental wild-type strain, compared to the RefSeq sequences (NZ_CP009977.1 and NZ_CP009978.1). One of these mutations is in the *rpoS* gene that encodes the primary sigma factor of stationary-phase genes. Nine are found in the *cpsA* region encoding a sugar transferase that is annotated to be involved in the formation of extracellular sugar polymers (lipopolysaccharide [LPS] or capsules). LPS are often targets of phages as initial attachment points (37). SNPs in *cpsA* may explain why phage spot assays in our hands never produced a yield in plaques (see the supplemental material for information on reinfection studies). Consequently, these mutations were also present in the derived prophage-free variants (Table S3). A few further sequence variations (1 to 2 per strain) were identified in the phage-free variants, and a complete overview is provided in Table S3.

**FIG 3:**
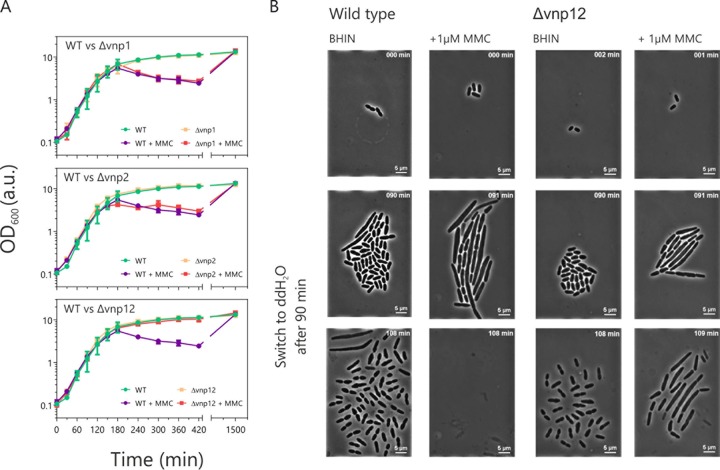
Prophage-free strains feature an increased tolerance against DNA-damaging conditions and hypo-osmotic stress. (A) The growth of the *V. natriegens* wild-type strain was compared with that of the phage-free variants (Δ*vnp1*, Δ*vnp2*, and Δ*vnp12* strains) in BHIN with or without 1 μM MMC in shaking-flask experiments. For each condition, three biological replicates of each strain were tested. Error bars represent the standard deviations. (B) Growth of the wild-type and Δ*vnp12* strains in microfluidic chip devices. Cells were grown in BHIN medium with 0.5 μM MMC for 90 min. To expose the cells to hypo-osmotic stress, the medium was changed to distilled water (ddH_2_O).

In a first set of experiments, we tested whether the removal of the proviral load and the few detected SNPs would affect the growth of the *V. natriegens* derivatives under various conditions. For this purpose, we used BHIN complex medium (see Materials and Methods) at 30°C and examined the growth in shaking-flask experiments. In our hands, no significant differences between the wild type and the prophage-free variants could be observed regarding growth rate and final optical density (OD) (Fig. 2B). Moreover, we extended the screening to a high-throughput approach conducted in 48-well plates. Here, we tested the growth of the strains on different carbon sources and monitored the impact of variations in pH, temperature, and osmolarity using VN minimal medium as a basis. Again, we did not observe any significant differences between the wild-type strain and the phage-free variants (Fig. 3B and Fig. S2).

Furthermore, we addressed whether the genome-wide expression profile was altered in the prophage-free strain by performing transcriptome sequencing (RNA-Seq) experiments. However, no significant changes in gene expression were observed between exponentially growing cells of the wild-type or the phage-free strains (*n* = 3; *P* value of <0.05 with a false discovery rate of 0.05; ArrayExpress accession no. E-MTAB-7877; see Materials and Methods), indicating the two prophages, VNP1 and VNP2, are very silent if the host grows exponentially in rich media. Moreover, the comparative characterization of the strains confirmed that the few additional SNPs (Table S3) do not negatively influence the growth of *V. natriegens* under the tested conditions.

Inspired by recent studies in which *V. natriegens* showed great potential as a platform strain for industrial biotechnology (24, 28), we compared the wild-type and the phage-free strains regarding their substrate consumption rates and the formation of glucose-derived products (such as acetate and pyruvate) (Fig. 2C). Remarkably, the prophage-free variant featured no significant differences in the biomass formation, glucose consumption rate, or acetate production under various sodium chloride concentrations (Fig. 2C). However, the pyruvate-per-glucose yield showed an increase of about 46% and 43% at a sodium chloride concentration of about 0.5% and 1.5% (wt/vol) g/liter, respectively (Fig. 2C).

### The prophage-free strain features an increased tolerance to DNA damage and hypo-osmotic stress conditions.

Under optimal growth conditions, we could not detect any negative impact of the prophage deletions on growth of *V. natriegens* (Fig. 2B). As a next step, we compared the behavior of the phage-free strains under stress conditions to that of the wild type. We first conducted a growth experiment in the presence and absence of the DNA-damaging antibiotic MMC (Fig. 3A). Here, the wild type and the Δ*vnp1* and Δ*vnp2* strains showed a drop in their optical density at 600 nm (OD_600_) after 3 h, indicating phage-dependent cell lysis. Interestingly, in the shaking-flask experiment, the Δ*vnp12* strain featured the same growth profile as cultures without the DNA-damaging agent. However, live-cell imaging experiments in microfluidic growth chambers confirmed that both the wild type and the prophage-free variant were affected by MMC and displayed a similar elongated cell morphology (Fig. 3B and Videos S1 and S2). This change in morphology is most probably a consequence of the activation of the cellular DNA damage response (SOS). Activation of the SOS response coincides with an induction of VNP1 and VNP2 in wild-type cells (as confirmed by sequencing experiments) (Fig. 1). In line with this assumption, wild-type cells showed a much higher susceptibility to hypo-osmotic stress than the phage-cured strain (Fig. 3B and Video S3). We addressed this phenotype in the microfluidic environment by changing the media from BHIN plus MMC to water after 90 min of cultivation (Fig. 3B and Video S3). A few minutes after the medium change, almost all wild-type cells lysed, whereas cells lacking both prophages remained intact (Fig. 3B). The likeliest reason for the increased sensitivity is the production of phage-encoded lysins or holins (e.g., PHASTER predicts a holin protein in the VNP1 region [Table S1]). These proteins degrade the peptidoglycan or form pores in the cell membrane and destabilize the host’s cell wall. A higher turgor pressure caused by a hypo-osmotic shock consequently will lyse cells producing these phage proteins. Taking these findings together, we can conclude that the prophage activation in combination with hypo-osmotic stress kills wild-type cells efficiently, whereas the majority of prophage-free cells stayed intact.

### Activity profile of VNP1 and VNP2.

**FIG 4:**
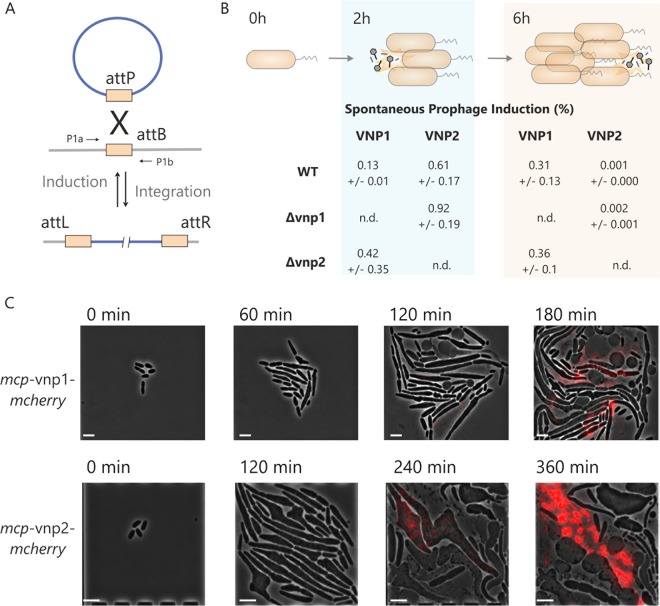
Spontaneous (A and B) and induced (B) activation of VNP1 and VNP2. (A and B) The spontaneous induction of the two prophages was addressed by qPCR using oligonucleotides designed for the quantification of genomes with an excised prophage element (VNP1 or VNP2) by amplifying the corresponding *attB* site. As a genomic control, oligonucleotides for a 194-bp product within the thymidine kinase gene (PN96_RS07840) were used. DNA from the phage-free strain served as a positive control (100% *attB*). Experiments were performed in triplicates (± standard deviations). Samples of the wild-type, Δ*vnp1*, and Δ*vnp2* cells were taken after 2 h (exponential phase) and 6 h (stationary phase). (C) In the Δ*vnp2* strain the MCP (encoded by PN96_RS04515) of VNP1 was fused to mCherry, and in Δ*vnp1* the VNP2 *mcp* gene (PN96_RS07045) was fused with the *mcherry* gene. The two reporter strains were cultivated in the microfluidic environment in BHIN with 1 μM MMC (Video S4). The phase contrast channel was merged with the fluorescence channel (excitation 560/40 nm; emission, 630/60 nm). Formation of fluorescent particles was visible within cells followed by cell lysis. The scale bar represents 5 μm.

Spontaneous prophage induction is a ubiquitously observed phenomenon of lysogenic bacterial strains (12). In the following experiments, we examined the fraction of cells undergoing SPI of VNP1 and VNP2 under standard cultivation conditions using quantitative PCR (qPCR) (Fig. 4A and B). For this purpose, genomic DNA was isolated from pelleted cells to avoid background signal of phage DNA enriching in the culture supernatant. The oligonucleotides were designed to anneal to the flanking regions of the host genome, yielding a product after the prophage excision (Fig. 4A). Overall, in this experiment, we determined that SPI is occurring in less than 1% of the cells of an exponentially growing WT population (0.13% ± 0.01% for VNP1 and 0.61% ± 0.17% for VNP2). Depending on the host’s growth phase, the SPI fraction of VNP1 was found to differ from that of VNP2 (Fig. 4B). In the exponential phase (after 2 h), the fraction of VNP2-induced cells was shown to be four times larger than the fraction of VNP1-induced cells. In contrast, a higher fraction of VNP1-induced cells was observed in stationary phase (0.31% ± 0.13%, in contrast to 0.001% ± 0.000% for VNP2) (Fig. 4B). Very similar trends were observed by a further qPCR analysis in which the formation of circular phage DNA was monitored (Fig. S4). Here, larger amounts of total VNP2 DNA were quantified in exponentially growing cells, whereas in the stationary phase a larger amount for VNP1 was detected (Fig. S4). Moreover, for VNP1 as well as for VNP2, 2- to 3-fold higher activities (SPI fractions and circular DNA amounts) were observed in the respective deletion strains (VNP1 in Δ*vnp2* strain and VNP2 in Δ*vnp1* strain) (Fig. 4B and Fig. S4).

PHASTER predicted VNP1 to be an incomplete phage that gave rise to the question of whether there is a dependency between the two phages. To test if cell lysis and phage particle formation can be conducted independently by VNP1 and VNP2, we constructed reporter strains for each phage. For this, we first labeled a major capsid protein (MCP) of each phage by the mCherry reporter protein and tested the strains after MMC induction by fluorescence microscopy. By this set-up, we successfully monitored *in vivo* the formation of either VNP1 in the Δ*vnp2* strain or VNP2 in the Δ*vnp1* strain by a live-cell imaging approach (Fig. 4C and Video S4). In line with this finding, we conducted transmission electron microscopy (TEM) analysis of supernatants from Δ*vnp1* and Δ*vnp2* samples and confirmed the presence of siphophages in both samples (Fig. S5). Taken together, the live-cell imaging approach as well as electron microscopy analysis pointed out that VNP1 as well as VNP2 do form phage particles and are able to lyse the host independently. Moreover, quantitative PCR studies revealed VNP2 to be more frequently induced during the exponential growth phase, whereas VNP1-induced cells could still be observed in the stationary phase.

### Who wins the race of competitive growth?

**FIG 5:**
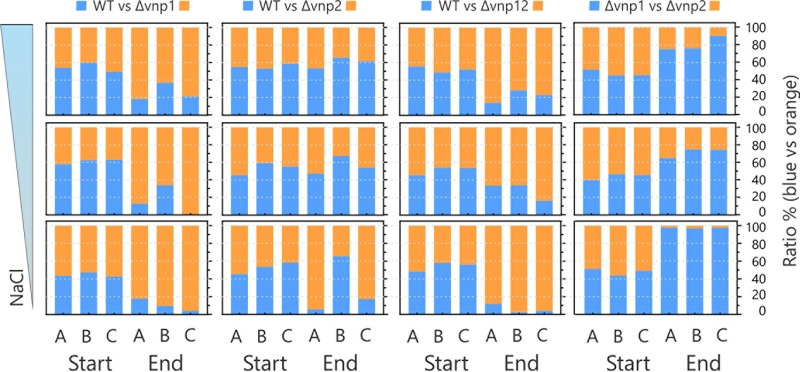
Prophage-free *V. natriegens* strain outperforms the wild type in a competitive growth experiment. Equal ratios of the indicated strains were cultivated in BHIN with 0.5%, 1.5%, or 2.5% (wt/vol) NaCl in a repetitive batch approach. Twice a day, stationary-phase cells were transferred to fresh medium (dilution, 1:200). The experiment was performed for 12 cycles (∼90 generations) in biological triplicates (A, B, and C). Culture composition was analyzed by qPCR. Shown is the composition of the initial cultures (cycle 1, ∼1:1) were compared with cells from the last batch (cycle 12).

Based on the fact that SPI causes a continuous loss of cells in a population, we assumed that the phage-free strain has an advantage when grown in direct competition with the wild type. To test this assessment, we conducted a competitive growth experiment in a repetitive batch approach. We used mixed cultures at a ratio of 1:1 under slightly hypo-osmotic (BHI plus 5 g NaCl·liter^−1^), standard (BHI plus 15 g NaCl·liter^−1^), and slightly hyperosmotic (BHI plus 25 g NaCl·liter^−1^) conditions. The experiment lasted for 90 generations, and the respective final culture composition (strain-to-strain ratio) was addressed by qPCR. Interestingly, the prophage-free Δ*vnp12* strain outcompeted the wild type under all tested conditions (Fig. 5). In contrast, the strain without the VNP2 prophage was only able to outcompete the wild type under low-salt conditions and only in two of three experimental runs, whereas the Δ*vnp1* strain outcompeted the WT strain under all tested conditions (Fig. 5). In a fourth comparison we directly tested the Δ*vnp1* versus the Δ*vnp2* strain. In this setting, the absence of VNP1 apparently conferred a fitness advantage to the Δ*vnp1* strain, which outcompeted the Δ*vnp2* strain under all tested conditions (Fig. 5).

## DISCUSSION

In this study, we report on the construction and comparative characterization of a prophage-free variant of *V. natriegens* ATCC 14048. While showing no significant phenotypic drawback in multiple experiments, the prophage-free variant was able to outcompete the wild type in a competitive growth experiment. In addition, the prophage-free strain appeared to be more robust under DNA-damaging and hypo-osmotic stress conditions. This is particularly interesting, as a reduction of the salt dependency will remain a major challenge for future efforts targeting the establishment of *V. natriegens* as a host for molecular biology and biotechnology (24, 28).

Lysogeny is common in nature (38), and although prophages are considered “molecular time bombs,” they contribute strongly to horizontal gene transfer (39) and the balance of microbial population dynamics (40, 41). However, considering the recent reports on the generation of prophage-free strains, domesticated laboratory strains apparently feature a more robust behavior when freed from their proviral load and therefore represent valuable platforms for metabolic engineering (as was shown for Pseudomonas putida, Corynebacterium glutamicum, and Lactococcus lactis) (17, 19, 21, 23). However, the metabolic costs for lysogeny are typically kept very low by the activity of so-called xenogeneic silencing proteins (42, 43). In *Proteobacteria*, H-NS-like proteins bind to AT-rich, horizontally acquired regions, forming extended nucleoprotein complex and thereby silencing foreign gene expression (44). By this mechanism, the bacterial host takes control over strong phage promoters and reduces the costs for unnecessary protein synthesis in the lysogenic life cycle of the phage. Therefore, the rather minor impact on growth observed in this study is not surprising and is in line with previous findings in C. glutamicum (18, 23) and P. putida (21).

Due to SPI, the presence of an inducible phage island will nevertheless lead to a continuous biomass loss during cultivation (12). SPI represents a well-known phenomenon of lysogenic bacterial strains but typically occurs in a small fraction of cells (<1%) depending on the strain background and growth conditions (45, 46). In this study, we harnessed mCherry fusions with the phage major capsid proteins (MCP) of VNP1 and VNP2 to monitor the induction process of each phage *in vivo*. This approach revealed that each phage alone indeed is capable of lysing the host cell upon induction by MMC (Fig. 4C). Quantification of SPI by qPCR revealed that the VNP2 prophage is more active in the exponential phase, whereas VNP1 still excised after the host has entered the stationary phase (Fig. 4B; see also Fig. S4B in the supplemental material). Remarkably, if one of the prophages was deleted, the remaining one exhibited a higher activity. The likeliest explanation is a cross talk of two *trans*-acting phage repressors in a way similar to what was reported for *Salmonella* prophages (47). This is supported by the fact that the two CI-type regulator proteins (VNP1, PN96_RS04515; VNP2, PN96_RS07045) share a high identity of 35%, covering 98% of the whole protein sequence (based on a blastp run with standard parameters).

SPI may also serve as an explanation for why the phage-free strain outcompeted the wild type under noninducing conditions (Fig. 5). However, this raises the question of why the deletion of VNP1 is more beneficial than the deletion of VNP2 even though the absolute VNP2 SPI fraction is about 4 to 5 times higher in the exponential phase. A hypothesis can be derived from the SPI differences observed for VNP1 and VNP2 and the assumption that under exponential growing conditions the loss of a minor population over time is compensated. If VNP2 was deleted, under normal conditions no benefits were observable (Fig. 5), although this phage was shown to be triggered in a higher fraction of cells (Fig. 4B). Overall, the data from the competitive growth experiment indicated that the presence of VNP1 represents a higher fitness burden than VNP2. This can be attributed either to the activity of an unknown gene product of VNP1 or to a different activity profile, as indicated by the SPI measurements (Fig. 4B). This lower burden conferred by VNP2 might be a reason why a wider spread among *Vibrio* species is observed on the basis of comparative genome analysis (see Fig. S2 and S3 in the supplemental material).

Remarkably, based on PHASTER predictions, most of the *V. natriegens* strains possess similar prophages (Fig. S2 and S3 and Table S2), and this raises the question of why they keep them. Typically, *Vibrio* species are halophilic bacteria that are able to take up DNA naturally. *V. natriegens* is no exception and is also capable of natural transformation (27). Consequently, harboring prophages causing stochastic release of DNA in *Vibrio* populations may contribute to horizontal gene transfer among the species.

Within the last 2 years, several independent studies highlighted the potential of *V. natriegens* as a host for small-molecule production (24), for cloning purposes (28), and as an interesting system for cell-free protein synthesis (29, 48). Furthermore, different tools for efficient genome engineering of *V. natriegens* have already been established (27, 28). In a recent study, Hoffart et al. engineered *V. natriegens* toward the production of the amino acid l-alanine, resulting in a strain with unprecedented volumetric productivity of about 34 g liter^−1^ h^−1^ (24). In this context, the increased pyruvate production observed in the prophage-free strain (>40% compared to that of the wild type) might represent a promising basis of future metabolic engineering efforts for the synthesis of pyruvate-derived products. In conclusion, our results emphasize the prophage-free strain of *V. natriegens* as a promising platform strain for future metabolic engineering featuring even further enhanced growth and genetic robustness compared to the wild-type strain.

## MATERIALS AND METHODS

### Bacterial strains and growth conditions.

All bacterial strains used in this work are listed in Table 1. Escherichia coli cells were propagated in liquid LB-Miller medium (10 g/liter tryptone, 5 g/liter yeast extract, 10 g/liter NaCl) or on agar plates (LB plus 15 g/liter agar [Carl Roth, Karlsruhe, Germany]) at 37°C. Chloramphenicol (34 μg/ml) was added to the medium, if not indicated otherwise. *V. natriegens* strains were cultivated in LBN (LB medium with 15 g/liter NaCl in total), BHIN (37 g/liter brain heart infusion [Becton, Dickinson, Franklin Lakes, NJ] plus 15 g/liter NaCl) or in VN medium (24) [per liter, 21 g 3-(*N*-morpholino)propanesulfonic acid (MOPS), 5 g (NH_4_)_2_SO_4_, 15 g NaCl, 1 g, KH_2_PO_4_, 1 g K_2_HPO_4_, 0.25 g MgSO_4_, 0.01 g CaCl_2_, 16.4 mg FeSO_4_ × 7 H_2_O, 10 mg MnSO_4_ × H_2_O, 0.3 mg CuSO_4_ × 5 H_2_O, 1 mg ZnSO_4_ × 7 H_2_O, 0.02 mg NiCl_2_ × 6 H_2_O, pH 7.5]. For agar plates, 15 g/liter agar was added with, if required, 15 μg/ml chloramphenicol. Cultivations were performed in test tubes (precultures) and shaking flasks (main cultures) or in 96-well deep-well plates (VWR, Radnor, PA) (for precultures) and 48-well Nunclon delta surface plates (Thermo Fisher Scientific, Waltham, MA) (main cultures for plate reader assays). For shaking-flask experiments, single colonies were used to inoculate 4 ml of BHIN medium in a test tube that was incubated on a rotary shaker at 180 rpm and 30°C for 2 h. Cells then were harvested from precultures, and the cell pellets were resuspended in the appropriate medium to an OD_600_ of 0.1 (if not indicated otherwise). Strains were cultivated at 30°C at a shaking frequency of 120 rpm. For growth assays performed in the plate reader (Infinite M1000; Tecan, Zürich, Switzerland), single colonies were picked and precultivated in 750 μl BHIN medium for 2 h at 30°C in 96-well deep-well plates at 900 rpm in an HT Microtron shaker (Infors HT, Bottmingen, Switzerland). Subsequently, the precultures were used to inoculate the main culture in 48-well plates, starting with an OD_600_ of 0.1. The temperature was set to 30°C, the shaking frequency was 582 rpm (orbital mode; amplitude of 1), and the optical density at 600 nm was measured online in 10-min intervals.

**TABLE 1:** Strains and plasmids used in this work

Strain or plasmid	Relevant characteristics/genotype	Reference or source
Strains		
Escherichia coli		
S-17 λpir	*thi pro hsdR hsdM*^+^ *recA* RP4-2-Tc::Mu-Km::Tn*7* λpir	66
Vibrio natriegens		
ATCC 14048	Referred here as the WT strain, DSM 759	DSMZ
Δ*vnp1*	VNP1 (PN96_RS04340–PN96_RS04575) was deleted, *attB* site was left intact	This work
Δ*vnp2*	VNP2 (PN96_RS06975–PN96_RS07190) was removed from the chromosome after integration of the *ccdB* toxin, excision of VNP2 left an intact *attB* site	This work
Δ*vnp12*	VNP regions 1 and 2 were deleted	This work
*Δvnp1*::*mcp-vnp2-mcherry*	In the Δ*vnp1* strain the *mcherry* reporter gene was fused to *mcp* of VNP2	This work
Δ*vnp2::mcp-vnp1-mcherry*	In the Δ*vnp1* strain the *mcherry* reporter gene was fused to *mcp* of VNP1	This work
Plasmids		
pDM4	*sacBR*; *oriT*; *ori* R6K; *CmR*	67
pDM4-del-vnp1	Deletion of VNP1 by homologous recombination and *sacB*-based counterselection	This work
pDM4-vnp2-pBAD-ccdB	Curing of *V. natriegens* from VNP2 by integrating the *ccdB* toxin into the VNP2 region	This work
pDM4-mcp-vnp1-mcherry	*mcherry* gene was fused to the *mcp* gene (PN96_RS04515) of VNP1 with the linker sequence GL(GSGG)_3_TA	53 and this work
pDM4-mcp-vnp2-mcherry	Fusion of the *mcherry* gene to the *mcp* gene (PN96_RS07045) of VNP2 with the linker sequence GL(GSGG)_3_TA	53 and this work

### Recombinant DNA work and chromosomal recombination.

Plasmids and oligonucleotides used in this work are listed in Tables 1 and 2. Routine cloning work, like PCR, plasmid restriction, DNA purification, etc., was conducted according to established protocols (49). For colony PCR with *V. natriegens* cells, single colonies were resuspended in 50 μl double-distilled H_2_O and heated for 10 min at 95°C. Subsequently, samples were briefly centrifuged (10 s), and 1 μl of the supernatants was used as the genomic DNA template for the PCR. To construct plasmids, purified PCR products and digested plasmid DNA were assembled according to the Gibson assembly protocol (50). Oligonucleotide synthesis and sequencing of plasmids were performed at Eurofins MWG Operon (Ebersberg, Germany).

**TABLE 2:** Oligonucleotides used in this work

Oligonucleotide	Sequence^*a*^ (5′–3′)	Comment
vnp1_del1	**AATTTGTGGAATCCCGGGAG**GCCGATGAGTCAGGCTCG	Deletion of VNP1 via homologous recombination
vnp1_del2	ACCACACAAATCTACACTCACGTTATC	
vnp1_del3	**TGAGTGTAGATTTGTGTGGT**TTTTACATCTGAGTG	
vnp1_del4	**CACTAGTGGGGCCCTTCTAG** GTTCCGGGCTTCGCTTAATGG	
vnp1_del5	GCCGGTACCAGAAGAAGACTTAG	
vnp1_del6	CGTATTAACACGAGACGAGATTCG	
araC_fw	**TCTGTGAAGAATATTAGTTC**TTATGACAACTTGACGGCTACATCATTC	Deletion of VNP2 via counterselection by integrating the toxin *ccdB* between PN96_RS07160 and RS07165
Pbad_rv	CGTTTCACTCCATCCAAAAAAACGGG	
ccdB_fw	**TTTTTGGATGGAGTGAAACG**ATGCAGTTTAAGGTTTACACCTATAAAAGAG	
ccdB_rv	**CTTTCAATCAAGGTTTTATC**TTATATTCCCCAGAACATCAGGTTAATGG	
LF_vnp2_fw	**AATTTGTGGAATCCCGGGAG**CTGTAATTAAGGAACTGTGTGCAATGAAC	
LF_vnp2_rv	GAACTAATATTCTTCACAGAATGTTCGAAG	
RF_vnp2_fw	GATAAAACCTTGATTGAAAGCATTAATACAAG	
RF_vnp2_rv	**CACTAGTGGGGCCCTTCTAG**CACCAAGTAGGATTCTTTGTCATTGG	
vnp2_del_fw	GATCGAGGCAATCGCAAGTTTAACC	
vnp2_del_rv	GCGATAACGTCAGCCATGGC	
mcp_vnp1_LF_fw	**AATTTGTGGAATCCCGGGAG**GCAGATTGTGCGCGAAGACATG	Construction of *mcp-mcherry* fusion for VNP1 and VNP2
mcp_vnp1_LF_rv	**CCTCCACCAGAGCCGAGACC**TGCCGCTGGGACACCTTTC	
mcp_vnp1_RF_fw	**GCATGGACGAGCTGTACAAGTAA**CCAAACCGTGATTTTCATTAATTAATGCC	
mcp_vnp1_RF_rv_n	**CACTAGTGGGGCCCTTCTAG**CTTCTATGATTGGCGGTTGCGC	
mcp_vnp2_LF_fw	**AATTTGTGGAATCCCGGGAG**CTCCAGACGTCGAAATGTTAGCTC	
mcp_vnp2_LF_rv	**CCTCCACCAGAGCCGAGACC**CGCTGCTGGCTTTTTGCCTAAAC	
mcp_vnp2_RF_fw	**GCATGGACGAGCTGTACAAGTAA**TTAATCCCAGTAACCATCTAAGCCAC	
mcp_vnp2_RF_rv	**CACTAGTGGGGCCCTTCTAG**GGCGGTCTAGTTCATTCATTTCAG	
mcherry_fw	**GGTCTCGGCTCTGGTGGAGGAAGTGGTGGAGGTTCTGGTGGCACTGCC**ATGGTGAGCAAGGGCGAGG	
mcherry_rv	TTACTTGTACAGCTCGTCCATGCC	
d_vnp1_qpcr_fw	GCGAACGCCATTAGTAATCTGTTG	Oligonucleotides used for qPCR analysis of the competitive growth experiments and to determine the fraction of SPI-positive cells
d_vnp1_qpcr_rv	CTCGAATCAGGTGCCGATATCAG	
d_vnp2_qpcr_fw	TCGGCTTTTTCATATTCACAACTTTACC	
d_vnp2_qpcr_rv	CATCTGTGCGAACACCAGCAAATTG	
WT_ctrl_tkin_fw	GGCACTCATCAACAAGAATACAATGTC	
WT_ctrl_tkin_rv	CTACCAAGAACGCGGCATGAC	
vnp1_circ_q_fw	GAAGATGGGTAATTATATGTGACGCG	
vnp1_circ_q_rv	TGAATTGTCCCACCAGCGCC	
vnp2_circ_q_fw	CGCTAATCGACTGATAAACAAGGATAG	
vnp2_circ_q_rv	GGGCGTCTTTTTTTGGTTGTTGTTTG	

a Boldface sequences represent the overlapping regions that are used for Gibson assembly.

Chromosomal deletion of prophage region 1 (positions 935757 to 971809; size, 36,052 bp) was done by homologous recombination with two ∼500-bp large flanking regions using the plasmid pDM4-del-vnp1 as described previously (51, 52). To construct pDM4-del-vnp1, first the 500-bp flanking regions were amplified by PCR using the oligonucleotides vnp1_del1 and vnp1_del2 for the upstream region and vnp1_del3 and vnp1_del4 for the 500-bp downstream region. PCR products were purified, and the backbone plasmid pDM4 was digested with SacI and XbaI, purified, and subsequently combined with PCR via Gibson assembly. For the transformation, E. coli S-17 λpir cells were used. Positive clones (evaluated by colony PCR) were sequenced and used for the conjugation protocol (51, 52). After the last counterselection step, clones were tested by colony PCR using the oligonucleotides vnp1_del5 and vnp1_del6. To remove prophage region 2 (1496626 to 1535809; size, 39,183 bp), the toxin *ccdB* under the control of P*_BAD_* was integrated between PN96_RS07160 and PN96_RS07165 using the pDM4-vnp2-pBAD-*ccdB* plasmid. The plasmid was constructed in a fashion similar to that described for pDM4-del-vnp1. Here, two ∼500-bp homologous flanking regions of the integration site (between PN96_RS07160 and RS07165) were used for the recombination and counterselection. After the 2nd recombination, the absence of prophage region 2 was confirmed by colony PCR (vnp2_del_fw and vnp2_del_rv). Note that the integration of the *ccdB* gene already resulted in the isolation of strains that spontaneously lost the VNP2 region. Finally, the deletions of both prophage regions were checked by genome sequencing (see below).

Fusion of the *mcherry* reporter gene to the *mcp* genes of the prophage regions 1 and 2 was achieved using the plasmids pDM4-mcp-vnp1-mcherry and pDM4-mcp-vnp2-mcherry, respectively. Here, the gene products result in a C-terminal fusion of the mCherry protein connected by a linker sequence [GL(GSGG)_3_TA] (53). For this purpose, the *mcherry* gene with the upstream linker sequence GGTCTCGGCTCTGGTGGAGGAAGTGGTGGAGGTTCTGGTGGCACTGCC was integrated in frame to the respective *mcp* gene (without a stop codon). Chromosomal integration was done by homologous recombination using ∼500-bp flanking regions of the integration site (as described above).

### Cultivation in microfluidic chip devices.

For growth experiments in the microfluidic environment, an in-house-developed platform was used as described in previous studies (54, 55). To enable proper growth of *V. natriegens* cells, the height of the growth chambers was set to be at least 1.15 μm. Phase-contrast and fluorescence imaging was conducted at the indicated time intervals using an inverted epifluorescence microscope (TI-Eclipse; Nikon, Duesseldorf, Germany). The medium flow was adjusted to 300 nl·min^−1^, and cells were cultivated at a constant temperature of 30°C. Cells from the exponential phase (OD_600_ between 0.1 and 0.5) were taken to load the cultivation chambers. To detect the mCherry signal, a Texas red filter (excitation, 560/40 nm; emission, 630/60 nm) was used in the fluorescence channel.

### Preparation of samples for TEM.

For TEM analysis, samples were prepared as described in Fortier and Moineau (56). Wild-type, Δ*vnp1*, and Δ*vnp2* cells were induced with 1 μM MMC at an OD of 0.1. Samples of 1 ml were taken after 3 h and 4 h and filtered (0.22-μm pore size).The filtrated phage samples were centrifuged at 16,000 × *g* for 1 h, supernatant was gently removed (∼900 μl), and 1 ml of ammonium acetate (0.1 M, pH 7.5) was added. This washing step was performed twice. The washed samples were allowed to adsorb on glow-discharged Formvar carbon-coated nickel grids (200 mesh; Maxtaform; Plano, Wetzlar, Germany) for 10 min. On the grids, the samples were stained by placing a drop of 0.5% (wt/vol) uranyl acetate in distilled water (Science Services GmbH, Munich, Germany). After air drying, samples were examined using a TEM LEO 906 (Carl Zeiss, Oberkochen, Germany), operating at an acceleration voltage of 60 kV. A wide-angle dual-speed 2K charge-coupled device camera (14 bit; Tröndle, TRS Moorenweis, Germany) and analysis software IMAGE SP Professional (SISPROG; Tröndle, Moorenweis, Germany) were used for imaging.

### Phage plaque and spot assays.

At an OD_600_ of 0.1 in BHIN, WT cells were induced with 1 μM MMC. After 3 h, 1 ml of the supernatant was taken (5 min, 16,000 × *g*) and filtered (0.22-μm pore size) and is referred to as the phage suspension. In parallel to the wild-type culture, single colonies of prophage-free variants (Δ*vnp1*, Δ*vnp2*, and Δ*vnp12* strains) were used to inoculate BHIN cultures (bait cultures) and were grown for at least 2 h at 30°C. For a standard plaque assay, 100 μl of the phage suspension was incubated with 800 μl bait culture and 100 μl 10× phage buffer (1× 10 mM Tris-HCl, pH 7.5, 10 mM MgSO_4_, 0.4% [wt/vol] NaCl) plus 2 mM CaCl_2_ for 20 min at room temperature. At this step several dilution series of the phage and cell suspension were tested. Subsequently, the phage-cell suspension was mixed with 4 ml prewarmed (water bath at 45°C) BHIN soft agar (0.5% [wt/vol] agar) and gently poured on a BHIN plate. After a cooling time of at least 1 h, plates were incubated at 30°C and in the following days visually checked for plaques.

In the spot assay, cell suspensions were directly mixed with the soft agar and poured on a BHIN plate. After approximately 1 h of cooling time, dilution series of the phage suspension (diluted with 1× phage buffer) were spotted on the top of the soft-agar plate. After a dry time of at least 30 min, plates were incubated at 30°C and inspected in the following days for plaques.

### Analytics for biomass yield, glucose consumption rate, and glucose-derived products.

The growth rate (μ [h^−1^]), the biomass yield (*Y_X/S_* [g g^−1^]), and the biomass-specific glucose consumption rate (*q_S_* [g g^−1^ h^−1^]) were calculated as described before (24). Biomass formation was monitored either by determining the OD_600_ or the cell dry weight (CDW; in g liter^−1^) at a given point in time (CDW [g liter^−1^] = OD_600_ × 0.27) (24). For determination of glucose and organic acid concentrations in the culture fluid, 2 ml of the culture was harvested by centrifugation (12,100 × *g*, 5 min, room temperature) and the supernatant was analyzed. Glucose and pyruvate concentrations were determined enzymatically according to reference 57. Other organic acid concentrations were measured via high-performance liquid chromatography using an Agilent 1200 series apparatus equipped with a Rezex ROA organic acid H^+^ (8%) column (300 by 7.8 mm; 8 μm; Phenomenex), protected by a Phenomenex SecurityGuard carbo-H column (height, 4 mm; inner diameter, 3.0 mm) as described by Hoffart et al. (24).

### Competitive growth experiment.

To analyze the fitness under competitive conditions, the wild-type strain was set up in an equal cell-to-cell ratio with the Δ*vnp1*, Δ*vnp2*, or Δ*vnp12* strain. Additionally, the Δ*vnp1* strain was cocultivated with the Δ*vnp2* strain. The competitive growth experiment was performed in 96-well deep-well plates at 30°C with a 900-rpm shaking frequency. Here, an OD_600_ of 0.05 for each test strain was adjusted (e.g., setup 1, wild-type and Δ*vnp1* strains; setup 2, wild-type and Δ*vnp2* strains, etc.) to inoculate an 800-μl culture (OD_600_ of 0.1) under standard conditions (BHI plus 1.5% [wt/vol] NaCl), minor hypo-osmotic stress (BHI plus 0.5% [wt/vol] NaCl), and minor hyperosmotic conditions (BHI plus 2.5% [wt/vol] NaCl). Twice a day, the final OD_600_ of stationary-phase cultures was measured, and 4-μl aliquots were transferred to inoculate a new 800-μl culture containing the appropriate medium (dilution, 1:200). The experiment lasted for 12 cultivation cycles. According to the equation OD_final_/OD_start_ = 2*^n^* (in which *n* is the number of generations), 90 generations were estimated in total. Approximately 700 μl of stationary-phase cells was taken after the first and last cultivation cycles. Cells were harvested (2 min, 10,000 × *g*) and pellets were stored at −20°C for further analysis.

### Sample preparation and qPCR analysis.

The culture compositions (fraction of the particular strains) in the competitive growth experiment as well as the fraction of SPI-positive cells were quantified by qPCR. In the SPI experiment, cells were taken from the exponential-phase cultures (2 h) and stationary-phase cultures (6 h) propagated in BHIN medium. Cell pellets were resuspended in the BE elution buffer provided by the NucleoSpin microbial DNA kit (Macherey-Nagel, Dueren, Germany), and the isolation of the genomic DNA (gDNA) was done according to the manufacturer’s protocol. The concentration of the gDNA was measured using a Qubit 2.0 fluorometer (Thermo Fisher Scientific, Waltham, MA) and adjusted to 50 ng/μl.

qPCR experiments were done using the Luna universal qPCR master mix (New England Biolabs, Ipswich, MA) and a qTOWER 2.2 instrument (Analytik, Jena, Germany) using 100 ng of the prepared gDNA as the template. The appropriate DNA standards were prepared after a PCR step with the oligonucleotides listed in Table 2 and a subsequent gel extraction with the NucleoSpin gel and PCR clean-up kit (Macherey-Nagel, Dueren, Germany). The concentration was measured with the Qubit 2.0 fluorometer and adjusted to 1 ng/μl. From this sample five 1:10 serial dilutions were prepared (1 × 10^−5 ^ng/μl), and 2-μl aliquots of these samples were used as standards (10^−3^ to 10^−5 ^ng/μl). All reactions were run in technical duplicates (*R*^2^ > 0.95). As the genomic reference region, 194 bp of the thymidine kinase-encoding gene from the PN96_RS07840 locus was used. For the competitive experiment, the genomic DNA of the prophage-free and the wild-type strains were taken as controls. The level of WT DNA was considered 100%, whereas the DNA of the phage-free strain was used as a negative control. The ratio was calculated using the following equation: ratio_Δstrain/WT_ = *N*(DNA_sample_)/*N*(DNA_WT_) × 100. In the SPI experiment, to calculate the fraction of cells featuring spontaneous prophage induction, the genomic DNA of the prophage-free strain was referred to as the fully induced (100%) control by using the following equation: SPI fraction (%) = *N*(DNA_sample_)/*N*(DNA_Δ_*_vnp12_*) × 100, where *N*(DNA_Sample_), *N*(DNA_Δ_*_vnp12_*), and *N*(DNA_WT_) are the normalized DNA amounts that were calculated using the measured threshold cycle values and appropriate DNA standards. The normalization was done to a genomic control gene (encoding a thymidine kinase): *N*(DNA*_X_*) = DNA*_X_* × DNA_reference_.

To determine the ratio of phage to bacterial genomes (see Fig. S6 in the supplemental material), the number of phage genomes that was calculated from the circular phage DNA amounts was divided by the number of the overall bacterial genomes obtained from the DNA amount using the reference gene. DNA of the phage-free strain was used as a negative control.

### Resequencing of Vibrio natriegens genomes and data analysis.

For sequencing of gDNA, isolation was performed using the NucleoSpin microbial DNA kit (Macherey-Nagel, Dueren, Germany). Seven hundred μl of stationary *V. natriegens* cells yielded approximately 20 μg DNA, of which 4 μg was used for the library preparation (see below). DNA from the supernatant was extracted by phenol-chloroform isoamyl alcohol (PCI) extraction, which was slightly modified according to established protocols (58). Supernatant samples were treated with 10% (wt/vol) sodium dodecyl sulfate (SDS) at a ratio of 5:1 and 1 mg/ml proteinase K (Macherey-Nagel, Dueren, Germany) for 1 h at 65°C. Subsequently, an equal volume of PCI was added to the treated supernatant, gently mixed, and transferred into phase-lock tubes (Quantabio, Gaithersburg, MD). After phase separation, DNA from the liquid phase was precipitated by ethanol (with 3 M sodium acetate). DNA concentration was measured using the Qubit 2.0 fluorometer. Again, 4 μg of DNA was used for the library preparation that was conducted with TruSeq DNA PCR-free (Illumina, San Diego, CA). Libraries were evaluated by qPCR with the KAPA library quantification kits (Peqlab, Erlangen, Germany) and subsequently normalized for pooling. Paired-end sequencing was performed with a MiSeq sequencing device (Illumina, San Diego, CA) with a read length of 2× 150 bases, and output (base calls) data were stored as demultiplexed fastq files. Data processing, such as trimming, mapping, and extracting the coverage sequences, was performed with the CLC Genomics Workbench (Qiagen Aarhus A/S, Aarhus, Denmark). For the mapping, as reference sequences the RefSeq replicon entries NZ_CP009977.1 and NZ_CP009978.1 of the *V. natriegens* strain ATCC 14048 were taken. Variant analysis was done by uploading the demultiplexed raw reads to PATRIC (59) and applying the variation analysis with BWA-mem as the aligner and FreeBayes as the SNP caller. Data visualization was done with R (60). The obtained reads were uploaded to ArrayExpress and are available under the accession number E-MTAB-7875.

### Analysis of differential gene expression (RNA-Seq).

Starting from an OD_600_ of 0.1 in BHIN, wild-type cells and the three prophage-free variants (Δ*vnp1*, Δ*vnp2*, and Δ*vnp12* strains) were grown at 30°C for 2 h. Cells were harvested by centrifugation (10 min at 4,500 × *g*, 4°C). The pellets were immediately frozen in liquid nitrogen. RNA extraction was conducted using the RNeasy kit (Qiagen, Hilden, Germany) as described previously (45). The quality of the obtained RNA was first evaluated in an Agilent Tape station 2200 (Agilent Technologies, Waldbronn, Germany) by assessing the RNA integrity number (RIN). If the RIN values were above 8.0, the RNA was depleted for ribosomal amounts with the Ribo-Zero rRNA removal kit (bacteria) (Epicentre/Illumina, Munich, Germany). Subsequently, the depletion was verified by a further capillary-based electrophoresis. For two of the three biological replicates the cDNA library was prepared with the TruSeq stranded emRNA library preparation kit (Illumina, Munich, Germany), and for one biological replicate of each strain the NEBNext Ultra RNA library preparation kit for Illumina (NEB, Ipswich, MA) was used. Validations of the libraries were performed by qPCR either using the KAPA library quant kits (Peqlab, Erlangen, Germany) for Illumina libraries or, for the NEB-based libraries, the NEBNext Library quantification kit for Illumina. Paired-end sequencing was conducted with the MiSeq reagent kit v3 (150-cycle) in a MiSeq platform (Illumina, Munich, Germany). Demultiplexed raw reads were uploaded to the ArrayExpress database at EMBL-EBI under the accession number E-MTAB-7877. To conduct the differential gene expression (DGE) analysis, first the raw reads were trimmed for quality and mapped against the genome sequence of the *V. natriegens* strain ATCC 14048 (RefSeq accession numbers NZ_CP009977.1 and NZ_CP009978.1) with the CLC Genomics Workbench (Qiagen Aarhus A/S, Aarhus, Denmark). For the alignment, only the reads that have a similarity of at least 90% in 90% of their lengths were considered. As a next step, the mapped reads were normalized using the transcript per million method. The DGE analysis was conducted with the CLC Genomics-implemented tool for empirical DGE analysis (EDGE) with a cutoff *P* value of 0.05 and a false detection rate of 0.05.

### Comparative genomics of VNP1 and VNP2.

Genomes for all 5,730 publicly available *Vibrio* sp. genomes were extracted from the SRA and assembled (January 2018). Genome assemblies are stored in Enterobase.warwick.ac.uk. Each genome was searched for prophages using the PHASTER API (34). For each genome the sequences of intact, incomplete, and questionable prophages were extracted. For rapid searching of all prophage elements, a MASH sketch of each prophage sequence was created using the following parameters: sketch -k 21 -s 1000 (61). Phages similar to those of VNP1 and VNP2 were searched for using Mash (61). The sequences of prophages similar to VNP1 and VNP2 were extracted and annotated using Prokka (62) with a custom database constructed from the proteins extracted from all phages (January 2018). Prophage sequences were compared using nucmer –maxgap = 500 –mincluster = 100. Clustering was performed and heatmaps were drawn using heatmaply (63). Average nucleotide identity (ANI) was calculated using autoANI (64).

### Data access statement.

All data supporting the findings of this study are included in the manuscript (or supplementary file) or can be provided by the corresponding authors upon request. The NGS data generated in this study were deposited in the ArrayExpress database at EMBL-EBI (www.ebi.ac.uk/arrayexpress). Genome resequencing reads of the wild-type and prophage-free strains are accessible under E-MTAB-7875 (https://www.ebi.ac.uk/arrayexpress/experiments/E-MTAB-7875), and RNA-Seq reads of exponentially growing cells were deposited under E-MTAB-7877 (https://www.ebi.ac.uk/arrayexpress/experiments/E-MTAB-7877).

## Supplementary Material

Supplemental file 1

Supplemental file 2

Supplemental file 3

Supplemental file 4

Supplemental file 5

Supplemental file 6

Supplemental file 7

Supplemental file 8
