# Supplementary material for: Generation of a Prophage-Free Variant of the Fast-Growing Bacterium Vibrio natriegens
Source: Appl Environ Microbiol. 2019 Aug 14;85(17):e00853-19. doi: 10.1128/AEM.00853-19 (PMC6696956; doi:10.1128/AEM.00853-19)
Supplement: Supplemental file 1 [file AEM.00853-19-s0001.pdf]

Supplemental file to

Generation of a prophage-free variant of the fast-growing bacterium *Vibrio natriegens*

Eugen Pfeifer[1], Slawomir Michniewski[2], Cornelia Gätgens[1], Eugenia Münch[4], Felix Müller[4,5], Tino Polen[1], Andrew Millard[3], Bastian Blombach[5], and Julia Frunzke[1*]

1) Forschungszentrum Jülich GmbH, Institute for Bio- and Geosciences 1, IBG1, 52425 Jülich, Germany

2) Warwick Medical School, University of Warwick, Gibbet Hill Road, Coventry, CV4 7AL

3) Dept Genetics and Genome Biology, University of Leicester, University Road, LE1 7RH Leicester

4) Institute of Biochemical Engineering, University of Stuttgart, Allmandring 31, 70569 Stuttgart, Germany

5) Microbial Biotechnology, Campus Straubing for Biotechnology and Sustainability, Technical University of Munich, Straubing, Germany

* Corresponding authors

Prof. Dr. Julia Frunzke; j.frunzke@fz-juelich.de

Phone: +49 (2461) 61-5430

Supplementary Information

Susceptibility of *V. natriegens* Δvnp12 to VNP1 and VNP2 phage particles

As this aspect is of crucial relevance for the biotechnological application, we tested whether the prophage-free strains can be infected and lysed by the VNP1 and/or VNP2 phages. As described in the material and methods sections, we used phage-free variants as bait strains in plaque and spot assays with freshly purified VNP1 or VNP2 phage particles. In our hands, we could never observe the formation of phage plaques or spots under several tested conditions. In particular, we varied the concentration of $CaCl_2$ in the assay, the incubation temperatures (30°C & 37°C), growth phases of the baits strains, phage-to-cell ratio's, agarose concentrations in the two BHIN-agar layers and the incubation time for the infection. Neither of the conditions resulted in the appearance of clear or in turbid plaques. Based on these results, we assume that either the prophages are cryptic in terms of infectivity or the tested strains are resistant. In general, the isolation of *V. natriegens* phages is very similar to coliphages by performing classic plaque assays [exemplary shown in (1)]. VNP1 and VNP2 are both able to form particles resembling typical tailed icosahedral phage structures belonging to the family of *Siphoviridiae*. We cannot exclude that the two phages became cryptic by acquiring mutations hindering host infection (e.g. in spikes, tails, DNA injection proteins) under the tested conditions. Considering the re-sequenced genome of the host strain, it appears however to be more likely that the host strain became resistant. The genome re-sequencing of the wild type strain and its prophage-free variants revealed the presence of seven SNPs, ranging from several insertions to deletions, in the *cpsA* region in all strains (Table S3). The *cpsA* gene encodes a sugar transferase required for the synthesis of extra-cellular sugar-polymers referred as capsules or LPS. It is very likely, that these mutations are the cause for the resistance since extra cellular sugar polymers are common targets for the attachment by a phage and several studies have shown, that the loss of the capsule is accompanied with phage immunity (2).

Supplementary Figures

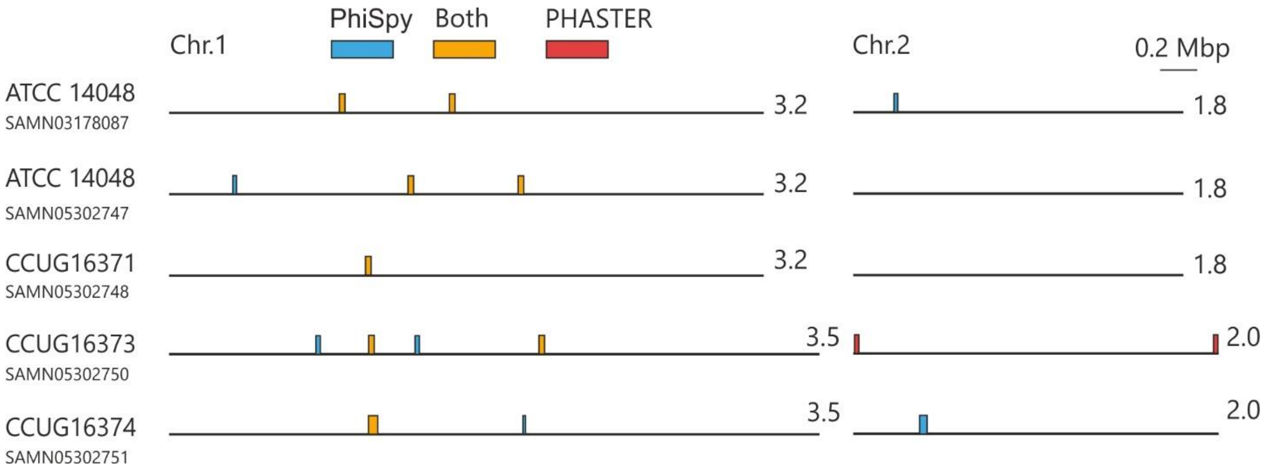

Figure S1: Prophages in different *Vibrio natriegens* isolates. Genomes of five different *Vibrio natriegens* strains of which complete sequences are available in the NCBI RefSeq database, were mined for prophages using PhiSpy (3) and PHASTER (4). This figure represents a schematic overview of the genomic positions from the prophage regions. Genome sizes of the bacterial chromosomes are indicated in mega base pairs (Mbp). Accession numbers of the BioSamples from the respective strains are given below the strain name. In supplementary Table S3 more detailed characteristics are given for the three predicted regions of the upper ATCC 14048 strain.

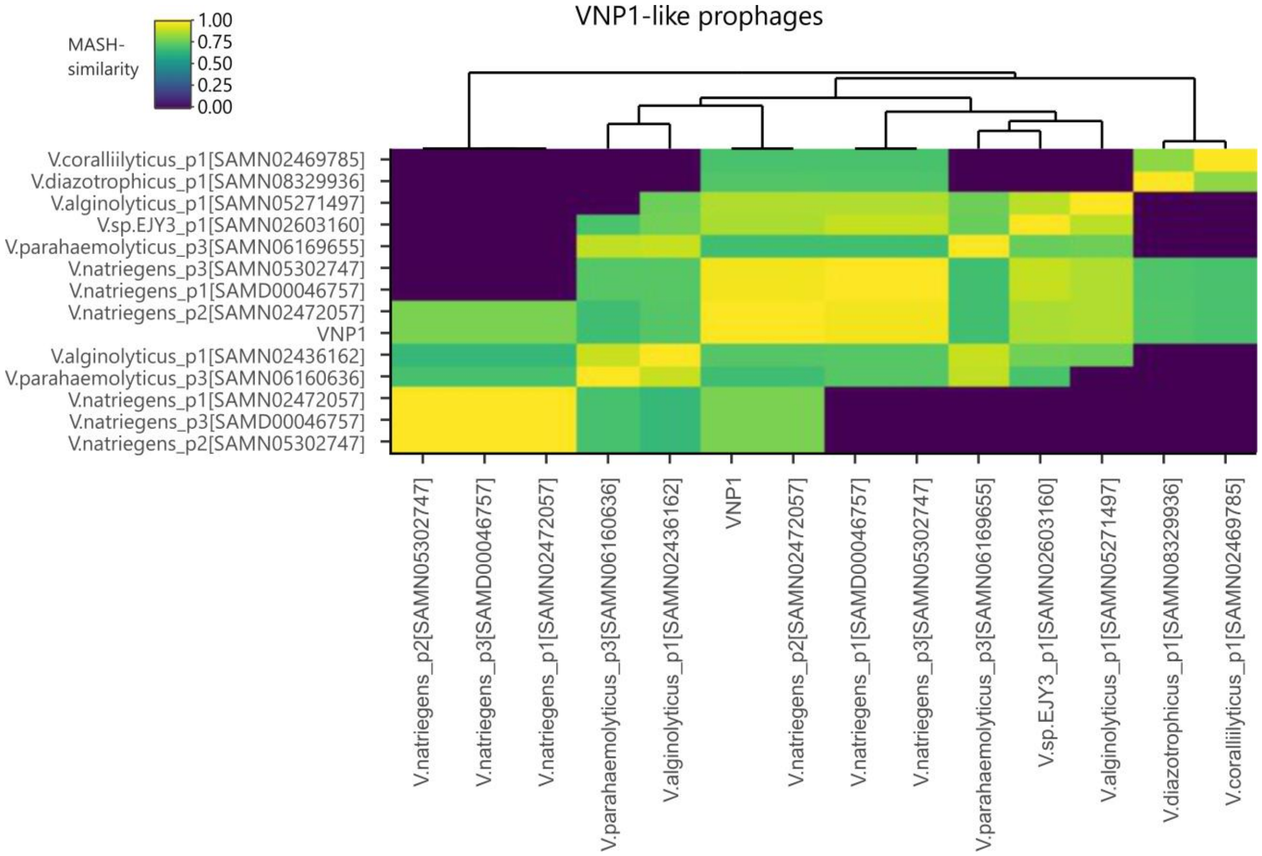

Figure S2: VNP1-like prophages in *Vibrio* species. A data base containing more than 10 000 PHASTER predicted prophages was screened for the presence of VNP1-like prophages. A hit was considered as positive if the MASH distances, that represent genome similarities, are lower than 0.3. Thirteen VNP1-like prophages were identified and hierarchically clustered using heatmaply (5).MASH values and further details are listed in suppl. Table S4. For clarity, only the column-based tree, obtained from clustering, is shown.

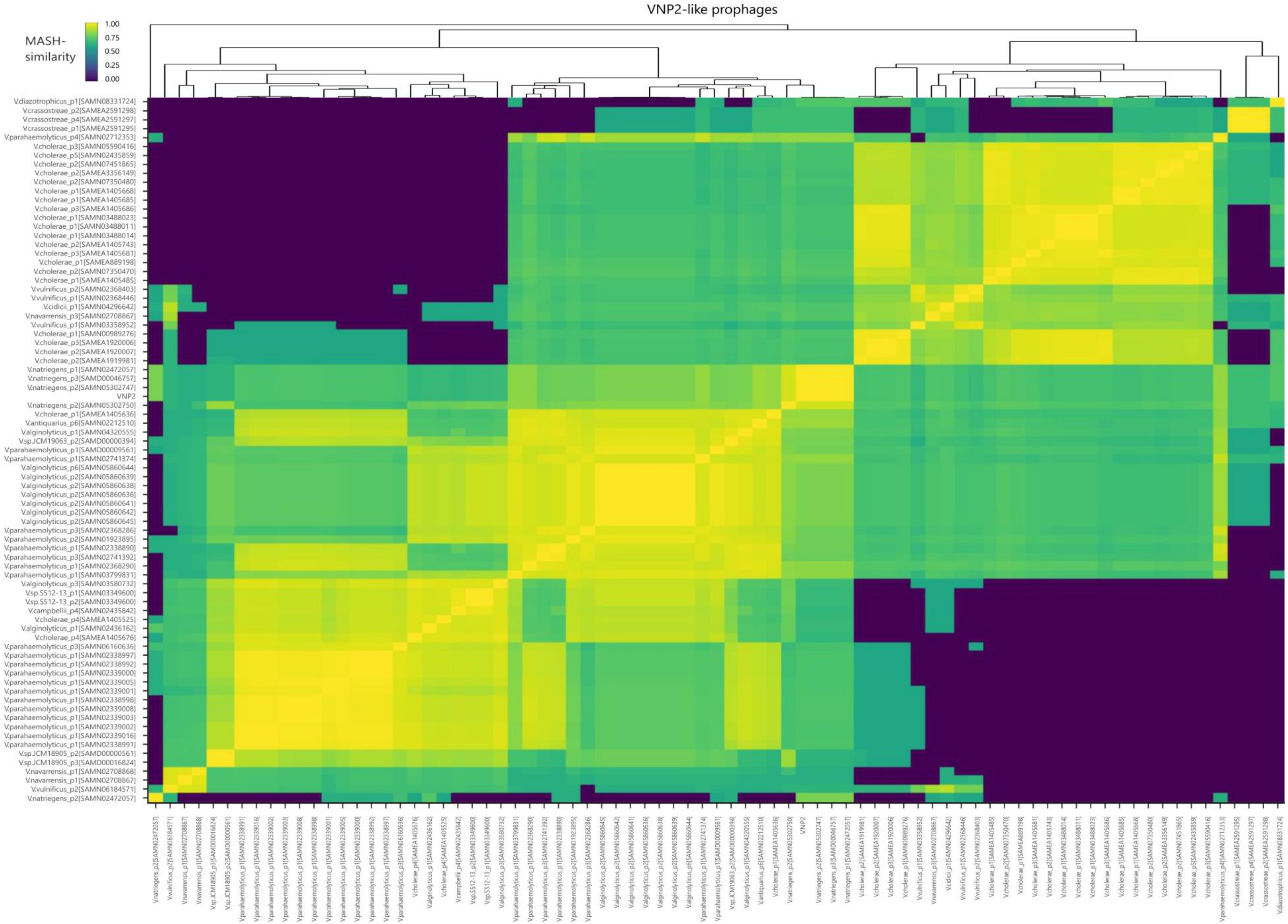
VNP2-like prophages
MASH-similarity
1.00
0.75
0.50
0.25
0.00
V.diazotrophicus_p1[SAMN08331724]
V.crassostreae_p2[SAMEA2591298]
V.crassostreae_p4[SAMEA2591297]
V.crassostreae_p1[SAMEA2591295]
V.parahaemolyticus_p4[SAMN02712353]
V.cholerae_p3[SAMN05590416]
V.cholerae_p5[SAMN02435859]
V.cholerae_p2[SAMN07451865]
V.cholerae_p2[SAMEA3356149]
V.cholerae_p2[SAMN07350480]
V.cholerae_p1[SAMEA1405668]
V.cholerae_p1[SAMEA1405685]
V.cholerae_p3[SAMEA1405686]
V.cholerae_p1[SAMN03488023]
V.cholerae_p1[SAMN03488011]
V.cholerae_p1[SAMN03488014]
V.cholerae_p2[SAMEA1405743]
V.cholerae_p3[SAMEA1405681]
V.cholerae_p1[SAMEA889198]
V.cholerae_p2[SAMN07350470]
V.cholerae_p1[SAMEA1405485]
V.vulnificus_p2[SAMN02368403]
V.vulnificus_p1[SAMN02368446]
V.cidicii_p1[SAMN04296642]
V.navarrensis_p3[SAMN02708867]
V.vulnificus_p1[SAMN03358952]
V.cholerae_p1[SAMN00989276]
V.cholerae_p3[SAMEA1920006]
V.cholerae_p2[SAMEA1920007]
V.cholerae_p2[SAMEA1919981]
V.natriegens_p1[SAMN02472057]
V.natriegens_p3[SAMD00046757]
V.natriegens_p2[SAMN05302747]
VNP2
V.natriegens_p2[SAMN05302750]
V.cholerae_p1[SAMEA1405636]
V.antiquarius_p6[SAMN02212510]
V.alginolyticus_p1[SAMN04320555]
V.sp.JCM19063_p2[SAMD00000394]
V.parahaemolyticus_p1[SAMD00009561]
V.parahaemolyticus_p1[SAMN02741374]
V.alginolyticus_p6[SAMN05860644]
V.alginolyticus_p2[SAMN05860639]
V.alginolyticus_p2[SAMN05860638]
V.alginolyticus_p2[SAMN05860636]
V.alginolyticus_p2[SAMN05860641]
V.alginolyticus_p2[SAMN05860642]
V.alginolyticus_p2[SAMN05860645]
V.parahaemolyticus_p3[SAMN02368286]
V.parahaemolyticus_p2[SAMN01923895]
V.parahaemolyticus_p1[SAMN02338890]
V.parahaemolyticus_p3[SAMN02741392]
V.parahaemolyticus_p1[SAMN02368290]
V.parahaemolyticus_p1[SAMN03799831]
V.alginolyticus_p3[SAMN03580732]
V.sp.S512-13_p1[SAMN03349600]
V.sp.S512-13_p2[SAMN03349600]
V.campbellii_p4[SAMN02435842]
V.cholerae_p4[SAMEA1405525]
V.alginolyticus_p1[SAMN02436162]
V.cholerae_p4[SAMEA1405676]
V.parahaemolyticus_p3[SAMN06160636]
V.parahaemolyticus_p1[SAMN02338997]
V.parahaemolyticus_p1[SAMN02338992]
V.parahaemolyticus_p1[SAMN02339000]
V.parahaemolyticus_p1[SAMN02339005]
V.parahaemolyticus_p1[SAMN02339001]
V.parahaemolyticus_p1[SAMN02338998]
V.parahaemolyticus_p1[SAMN02339008]
V.parahaemolyticus_p1[SAMN02339003]
V.parahaemolyticus_p1[SAMN02339002]
V.parahaemolyticus_p1[SAMN02339016]
V.parahaemolyticus_p1[SAMN02338991]
V.sp.JCM18905_p2[SAMD00000561]
V.sp.JCM18905_p3[SAMD00016824]
V.navarrensis_p1[SAMN02708868]
V.navarrensis_p1[SAMN02708867]
V.vulnificus_p2[SAMN06184571]
V.natriegens_p2[SAMN02472057]

Figure S3: Hierarchical clustering of VNP2-like prophages. Prophages that were predicted by PHASTER in 5730 genomes of *Vibrio* species were mined for VNP2-like prophages. Using a MASH distance of at least 0.3 over 77 VNP2-like prophages were identified. Based on the genome similarity (given by MASH values listed in suppl. Table S4) VNP2-like prophages were clustered using heatmaply (5). To provide a better overview, only the column-based distance tree is shown.

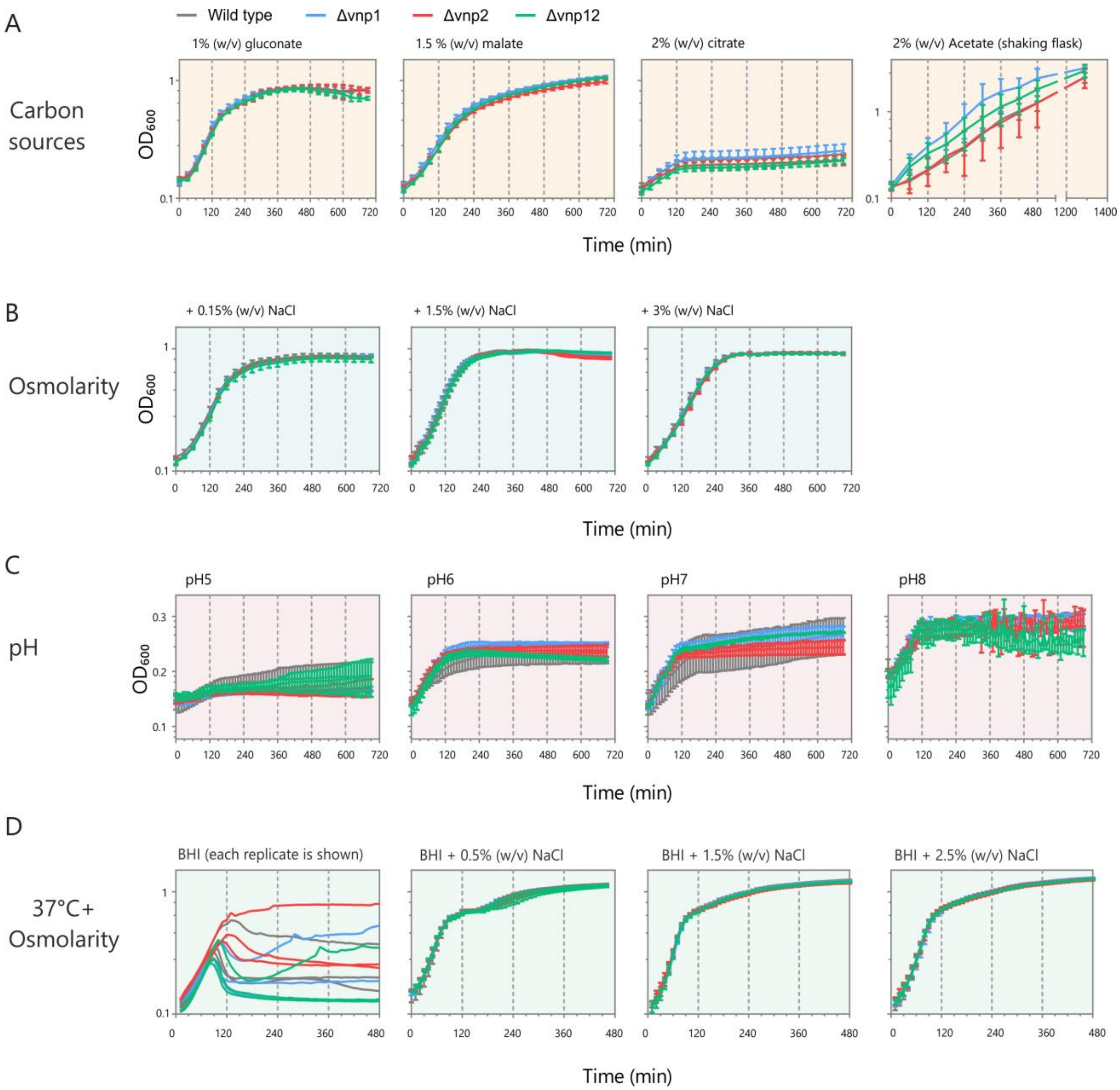

Figure S4: Comparative growth analysis of *V. natriegens* wild type vs phage-free strains. The growth dynamics of the *V. natriegens* wild type and its phage-free variants were compared in shaking flask and 48-well plate experiments. Shaking flask experiments were conducted if the pre-screen in the 48-well plate experiment has indicated a significant difference in growth under the tested conditions. Growth was followed by measuring the optical density at 600 nm every 30 min in shaking flask and every 10 min in the plate reader. Using VN media different carbon sources **(A)**, sodium chloride concentrations **(B)**, pH **(C)** and temperature conditions **(D)** were tested. Shown are mean values of three biological replicates and their standard deviations. In **(D),** at 37°C and a low sodium chloride concentration (in normal BHI media) strong fluctuations within the replicates of each strain were observed and thus each curve is shown. For each condition and strain three biological replicates were tested.

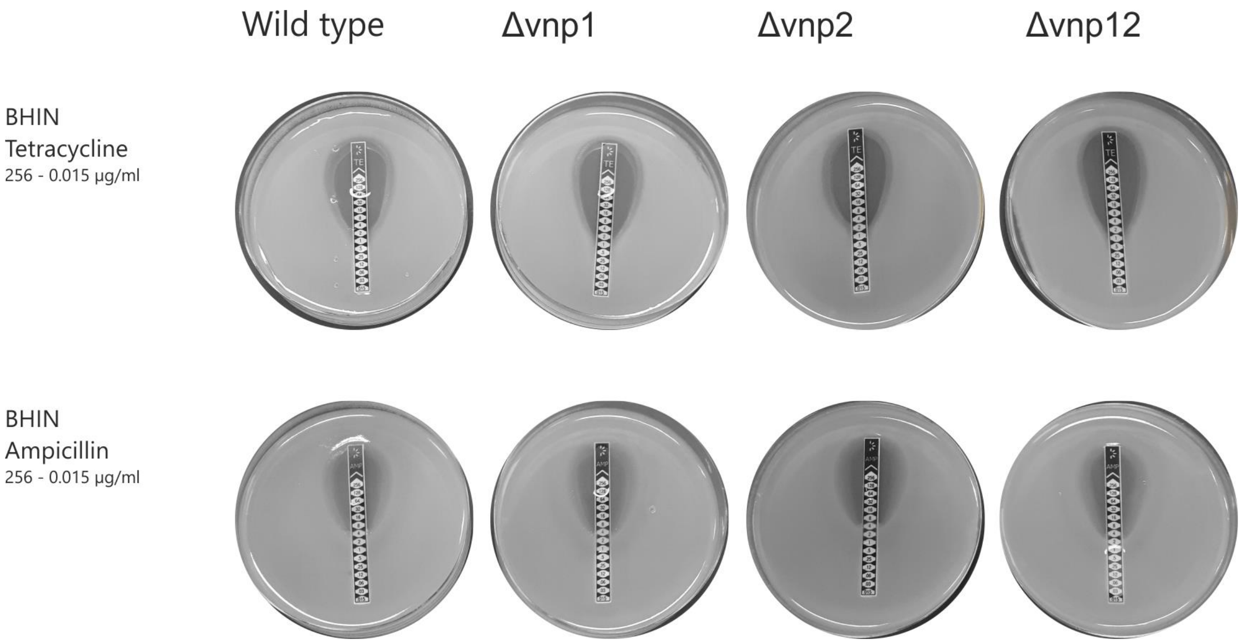

Figure S5: Comparative analysis of *V. natriegens* wild type and phage-free strains regarding their resistance towards selected antibiotics. Ampicillin and tetracycline antibiotic stripes (range: 256 – 0.015 µg·ml^{-1}) were applied on bacterial lawns from a BHIN top-agar assay to compare the growth and fitness of the wild type strain and its phage-free variants. Shown is one representative plate of each condition per strain of overall three biological replicates.

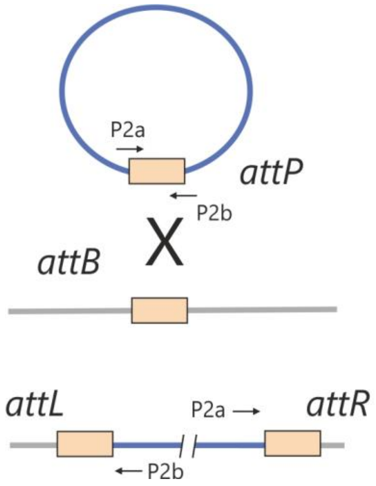

Phage genomes per bacterial genome

Strains	Exp. phase (2h) VNP1	Exp. phase (2h) VNP2	Stat. phase (6h) VNP1	Stat. phase (6h) VNP2
**WT**	0.014 +/- 0.007	0.059 +/- 0.034	0.009 +/- 0.004	0.002 +/- 0.000
**Δvnp1**	n.d.	0.173 +/- 0.020	n.d.	0.006 +/- 0.002
**Δvnp2**	0.028 +/- 0.025	n.d.	0.020 +/- 0.006	n.d.

Figure S6: Quantification of circular DNA of VNP1 and VNP2. The amount of circular VNP1 and VNP2 DNA within cells was measured by qPCR. As indicated in the left scheme, the oligonucleotides were designed to yield only in a product if the respective phage DNA is in its circular form. Shown are the ratios of n (circular phage DNA) per n (bacterial chromosomes). Samples for DNA isolation were taken after 2h (exp. phase) and 6h (stat. phase) of growth in BHIN at 30 °C. Mean values and standard deviations are based on three biological replicates for which two technical replicates were prepared for each strain and condition.

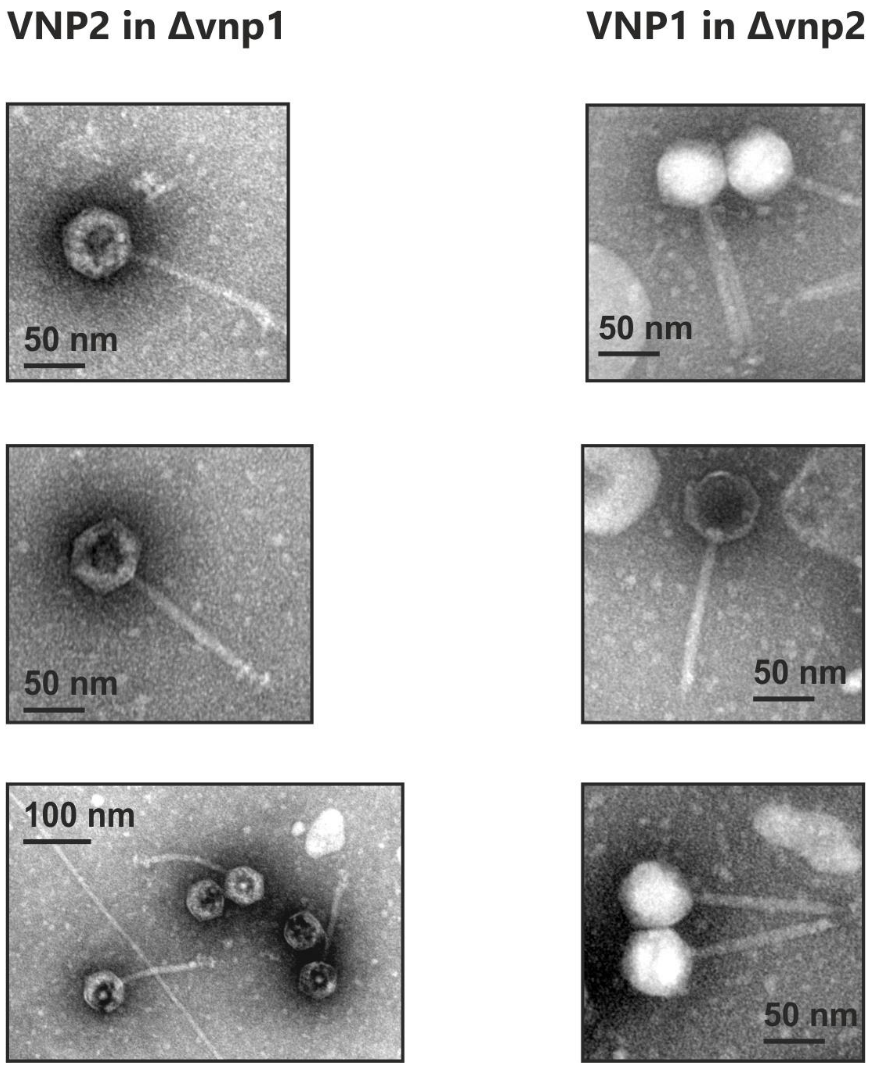

Figure S7: Phage particles produced by Δvnp1 and Δvnp2. Transmission electron microscopy analysis was conducted on supernatants of MMC-induced Δvnp1 and Δvnp2 strains. Supernatants were purified as described in the methods section. Shown are three representative images for each phage. Based on the morphology, VNP1 as well as VNP2 phage are classified as members of the *Siphoviridae.*

Legends to supplemental videos

Video S1. Growth of the wild type *Vibrio natriegens* strain ATCC 14048 was followed in microfluidic growth chambers in BHIN at 30°C.

Video S2. Wild type (left) and the Δvnp12 strain (right) were cultivated in BHIN at 30°C in the presence of 0.5 µM MMC.

Video S3. After wild type and prophage-free cells were induced by MMC (as shown in Video S2). After 90 min the medium was changed to distilled water to apply a hypo-osmotic stress.

Video S4. Phage reporter strains of which each is encoding a single integrated *mcp-mcherry* construct for VNP1 in Δvnp2 and for VNP2 in Δvnp1 were cultivated in the presence of 0.5 µM MMC.

References

1. Zachary A. 1976. Physiology and ecology of bacteriophages of the marine bacterium *Beneckea natriegens*: salinity. Appl Environ Microbiol 31:415-22.
2. Bertozzi Silva J, Storms Z, Sauvageau D. 2016. Host receptors for bacteriophage adsorption. FEMS Microbiol Lett 363.
3. Akhter S, Aziz RK, Edwards RA. 2012. PhiSpy: a novel algorithm for finding prophages in bacterial genomes that combines similarity- and composition-based strategies. Nucleic Acids Res 40:e126.
4. Arndt D, Grant JR, Marcu A, Sajed T, Pon A, Liang Y, Wishart DS. 2016. PHASTER: a better, faster version of the PHAST phage search tool. Nucleic Acids Res 44:W16-21.
5. Galili T, O'Callaghan A, Sidi J, Sievert C. 2018. heatmaply: an R package for creating interactive cluster heatmaps for online publishing. Bioinformatics 34:1600-1602.
